# Photoluminous Response of Biocomposites Produced with Charcoal

**DOI:** 10.3390/polym15183788

**Published:** 2023-09-16

**Authors:** Fabíola Martins Delatorre, Gabriela Fontes Mayrinck Cupertino, Allana Katiussya Silva Pereira, Elias Costa de Souza, Álison Moreira da Silva, João Gilberto Meza Ucella Filho, Daniel Saloni, Luciene Paula Roberto Profeti, Demetrius Profeti, Ananias Francisco Dias Júnior

**Affiliations:** 1 Department of Forestry and Wood Sciences, Federal University of Espírito Santo (UFES), Av. Governador Lindemberg, 316, Jerônimo Monteiro 29550-000, ES, Brazil; gabriela.mayrinck01@gmail.com (G.F.M.C.); 16joaoucella@gmail.com (J.G.M.U.F.); ananias.dias@ufes.br (A.F.D.J.); 2 Department of Forest Sciences, “Luiz de Queiroz” College of Agriculture, University of São Paulo (ESALQ/USP). Av. Pádua Dias, 11, Piracicaba 13418-900, SP, Brazil; allana.florestal@gmail.com (A.K.S.P.); alison.silva@usp.br (Á.M.d.S.); 3 Institute of Xingu Studies, Federal University of South and Southeast Pará (UNIFESSPA), Subdivision Cidade nova, QD 15, Sector 15, São Félix do Xingu 68380-000, PA, Brazil; eliascosta@unifesspa.edu.br; 4 Department of Forest Biomaterials, College of Natural Resources, North Carolina State University, Raleigh, NC 27695, USA; desaloni@ncsu.edu; 5 Department of Chemistry and Physics, Federal University of Espírito Santo (UFES), Alegre 29500-000, ES, Brazil; luciene.profeti@ufes.br (L.P.R.P.); demetrius.profeti@ufes.br (D.P.)

**Keywords:** biomass and biomaterials, uv-c radiation, mechanical properties, carbonaceous materials

## Abstract

Due to the possible effects of global warming, new materials that do not have a negative impact on the environment are being studied. To serve a variety of industries and outdoor applications, it is necessary to consider the impact of photoluminosity on the performance of biocomposites in order to accurately assess their durability characteristics and prevent substantial damage. Exposure to photoluminosity can result in adverse effects such as discoloration, uneven surface, loss of mass, and manipulation of the intrinsic mechanical properties of biocomposites. This study aims to evaluate general charcoal from three pyrolysis temperatures to understand which charcoal is most suitable for photoluminosity and whether higher pyrolysis temperatures have any significant effect on photoluminosity. Porosity, morphology, Fourier transform infrared spectroscopy (FTIR), and X-ray photoelectron spectroscopy of charcoal were analyzed. Charcoal obtained at a temperature of 800 °C demonstrates remarkable potential as a bioreinforcement in polymeric matrices, attributable to its significantly higher porosity (81.08%) and hydrophobic properties. The biocomposites were characterized for flexural strength, tensile strength, scanning electron microscopy (SEM), FTIR, and x-ray diffraction (XRD). The results showed an improvement in tensile strength after exposure to photoluminosity, with an increase of 69.24%, 68.98%, and 54.38% at temperatures of 400, 600, and 800 °C, respectively, in relation to the treatment control. It is notorious that the tensile strength and modulus of elasticity after photoluminosity initially had a negative impact on mechanical strength, the incorporation of charcoal from higher pyrolysis temperatures showed a substantial increase in mechanical strength after exposure to photoluminosity, especially at 800 °C with breaking strength of 53.40 MPa, and modulus of elasticity of 4364.30 MPA. Scanning electron microscopy revealed an improvement in morphology, with a decrease in roughness at 800 °C, which led to greater adhesion to the polyester matrix. These findings indicate promising prospects for a new type of biocomposite, particularly in comparison with other polymeric compounds, especially in engineering applications that are subject to direct interactions with the weather.

## 1. Introduction

For generations, materials from non-renewable sources have been used across almost all industry sectors. However, its excessive use and improper waste management have resulted in alarming pollution and greenhouse gas emissions worldwide, affecting society and the environment [1,2,3,4]. For instance, petroleum-based plastics, the first plastic generation, are associated with several environmental issues. When they are incinerated, a vast amount of CO_2_, greenhouse gas emissions [5,6], and hazardous synthetic substances are released, contributing to global warming and the deterioration of living beings’ health [7]. Moreover, due to their non-biodegradability, they cause air, land, and water pollution, resulting in the death of wildlife, marine life, and avifauna [1,5,7,8,9].

In response to these environmental concerns, there exists an imperative need for cleaner, sustainable, renewable product sources that can be locally produced. Thus, with the environmental crisis faced in recent years, the search for products obtained from sustainable resources is causing the technical–scientific environment to focus its research and technologies on elaborating so-called “green materials”. Concerns about environmental safety, reduction in greenhouse gas emissions, biodegradability, and mitigation of solid waste have resulted in a boost in the production of materials that come from waste and, at the same time, are renewable by nature [10,11,12]. This action includes the so-called industrial symbiosis, where waste from one sector becomes a resource for the generation of a product from another industry, effectively contributing to greater productivity of resources and favoring the circular economy [13,14,15]. To promote efficiency, innovation, and sustainability, several studies are investigating the combination of renewable waste with other materials of synthetic origins, such as polymers, resulting in the generation of composite materials with more sustainable characteristics [11,16,17,18,19].

Although ongoing research exists regarding the incorporation of charcoal as a reinforcing component in polymeric composites, there is a noticeable lack of detailed information on how the physicochemical properties of charcoal can influence the mechanical and thermal characteristics of biocomposites. When it comes to composite production, the consideration of their practical applicability is of paramount importance. Therefore, it is imperative to investigate the factors that can impact the strength and performance of these materials. Exposure to ultraviolet (UV) light plays a crucial role in the applications of polymeric materials in outdoor environments, as UV-induced degradation often significantly reduces the polymer’s lifespan [20]. Therefore, understanding how charcoal affects the resistance of the biocomposite to UV degradation is of paramount importance, particularly when these materials are intended for outdoor applications. In pursuit of developing innovative materials and meeting the demand for performance improvements throughout the product’s lifespan, it is crucial to conduct studies that investigate how photodegradation can influence a material’s properties. There are some reports in the literature about the increase in resistance to photoluminosity of some materials, such as asphalt, from the addition of charcoal [7,21,22,23]. However, the originality of this work lies in the generation of information on the influence of UV-c radiation on the properties of composites produced with charcoal synthesized from the waste of *Eucalyptus saligna* processing, a subject not found in the literature.

## 2. Materials and Methods

### 2.1. Obtaining Charcoal 

The charcoal came from the pyrolysis of *Eucalyptus saligna* wood from a ten-year-old experimental plantation. Samples of this biomass were fragmented in a Wiley knife mill and previously dried in an oven at 103 ± 2 °C. Pyrolysis was carried out in a metallic reactor inside a muffle furnace at an initial temperature of 30 °C heating rate of 10 °C.min^−1^ until reaching the final temperature of 400, 600, and 800 °C, with a residence time of 120 min. These procedures followed the descriptions by Dias Júnior et al. (2020) [24]. Then, to obtain the charcoal, samples of the obtained charcoal were fragmented in an MA-500 ball mill for three hours, and, subsequently, the material was sieved through a 250 mesh sieve (0.056 mm) to homogenize the samples. Finally, the charcoals were dried at 103 ± 2 °C in an oven.

### 2.2. Characteristics of Charcoal 

In order to understand how the characteristics of charcoal influence the biocomposites produced, the porosity was determined following the descriptions of the Brazilian Association of Technical Standards (ASMT) [25]. The evaluation of the structure of the material was determined from the Scanning Confocal Microscopy, with the aid of the Confocal Microscopic Olympus LEXT—3D Measuring L. Microscope 4000 (Evidente, Tóqui, Japão). The images were captured through an objective lens of 50× magnification (numerical aperture of 0.95, with a field of view of 0.26 mm × 0.26 mm and a sampling distance of about 0.25 µm) and a light beam length of 405 nm. For image processing, the OLS4000 2.1 software was used. To better understand the aromatic structures (functional groups and chemical bonds) of charcoal, a Fourier transform infrared spectroscopy (FTIR) analysis was performed in the Bruker, Ettlingen e Germany (Tensor 27 model), using a total attenuated reflectance (ATR) measuring the absorption of vibrations of the functional group in the mid-infrared region between 2500 and 15,400 nm (4000–650 cm^−1^) and acquisition with 32 scans.

The nature of C and O present on the surface of charcoal was determined using a K-Alpha spectrometer (Thermo Scientific), from the National Nanotechnology Laboratory (LNNano) of the National Center for Research in Energy and Materials (CNPEM, Campinas, Brazil), to carry out the spectroscopy analyses of X-ray photoelectrons (XPS). A monochromatic Al Kα X-ray source (λ = 1486.6 eV) with a power of 300 W, at a takeoff angle of 30° in relation to the surface of the samples, was used. Measurements took place under high vacuum of 5 × 10^−10^ mbar at room temperature. The investigated area was 81 mm^2^. The spectra were obtained in a binding energy range from 0 to 1150 eV, with three sweeps with a passing energy of 160 eV and a resolution of 1 eV, whereas the Gaussian peak profiles were used for spectral deconvolution of the C (1 s) spectral region.

### 2.3. Production of Biocomposites 

Silicone molds (bicomponent elastomer vulcanizable at room temperature) were made following the prescriptions of the dimensions of the flexion and traction tests. In the production of biocomposites, commercially obtained polyester resin from the Redecenter brand (São Paulo, Brazil) was used. Charcoals produced under different final pyrolysis temperatures (400, 600, and 800 °C) were previously dried in an oven (103 ± 2 °C) and used in proportions of 0 (control treatment) and 30%. In the process of homogenizing the charcoal with the polymeric matrix, a Fisatom 713DS mechanical stirrer (Fisatom, São Paulo, Brazil) was used, with a fixed time of three minutes or until homogenization was noticed. After mixing, the samples were taken to a metal reactor that operated under constant pressure at 90 KPa and room temperature (25 °C) for 24 h for the curing process.

### 2.4. Photoluminosity Analysis of Biocomposites 

**Figure 1:**
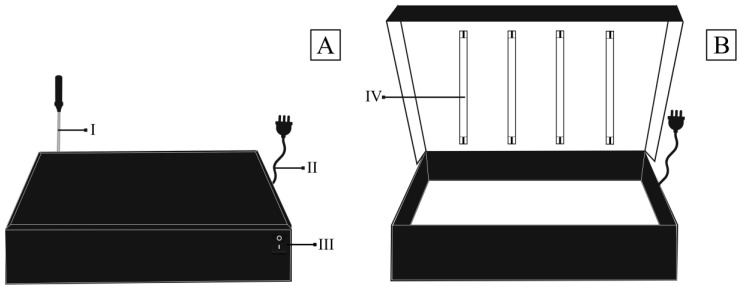
(**A**) front view of the photoluminosity box and (**B**) view of the inside of the photoluminosity box. Where: (I) thermocouple for measuring internal temperature, (II) switch, and (III) UV-c tubular fluorescent lamps.

The present invention refers to an innovative methodology for the treatment of photoluminosity in charcoal biocomposites, aiming to improve their properties and applications. The patent process number that protects this innovation is BR 10 2022 026841 0. Our approach revolutionizes the field of biocomposites by developing a photoluminosity chamber specially designed for this purpose, the concept of which is protected under intellectual property. Figure 1 shows the photoluminosity chamber used in the process, built with high-quality materials, notably MDF, and precisely sized to achieve optimal results. The photoluminosity chamber has strategic dimensions of 50 cm long, 15 cm wide, and 40 cm thick, providing a controlled environment for exposing the biocomposites to UV-c radiation. With four UV-c tubular fluorescent lamps, each with a power of 8 W, we guarantee uniform and effective irradiation, essential for the success of the methodology. The treatment process is conducted with scientific rigor, exposing the biocomposites to UV-c radiation for a continuous period of 15 days. During this time, the photoluminosity chamber remains hermetically closed, ensuring that no radiation or heat transfer takes place between the biocomposites and the external environment. This controlled condition is essential for obtaining reliable and reproducible results.

It is important to note that all tests were carried out in an environment under controlled conditions, with a constant temperature of 22 °C and relative humidity of 44%. This strict standardization of the environment guarantees that the results obtained are directly attributable to the action of the photoluminosity chamber on charcoal biocomposites. In conclusion, this innovation protected by patent BR 10 2022 026841 0 sets a new standard in improving the properties of charcoal biocomposites through photoluminosity. The specially developed chamber and controlled treatment conditions make this process invaluable, offering significant advances in the field of using biocomposite materials.

Both the composite (0% charcoal) and the biocomposite (30% charcoal) had characterization analyses performed before and after the photoluminosity analysis.

### 2.5. Characterization of Carbonaceous Biocomposites 

**Figure A1:**
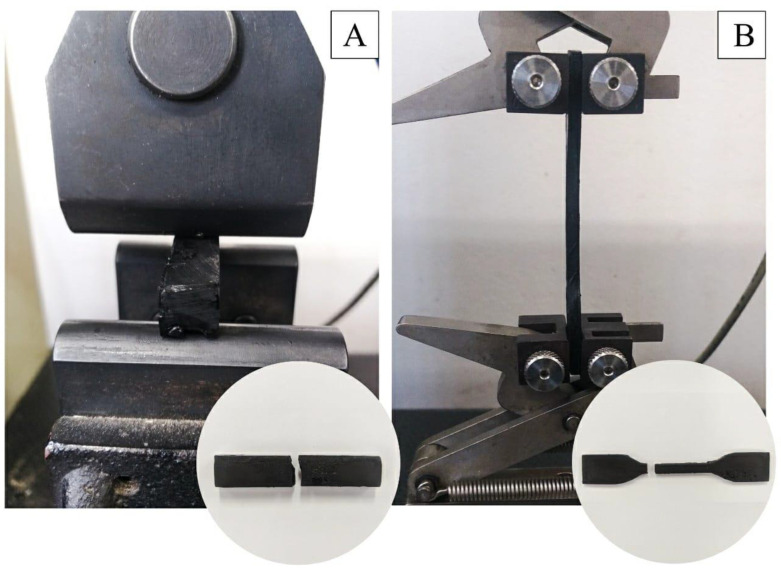
(**A**) flexural strength and (**B**) tensile strength of charcoal biocomposites.

Flexural and tensile tests were performed according to the standards of the American Society for Testing and Materials (ASTM) using the universal mechanical testing machine model EMIC. Flexural strength (Figure A1—Appendix A) was delimited following the parameters of the norm [26] and the test speed was maintained at 1 mm.min^−1^. The tensile tests followed the prescriptions of the standard [27] and stretching speed of 3 mm.min^−1^. In order to overcome experimental and instrumental errors, seven specimens were tested. For visualization of the structures in scanning electron microscopy (SEM), the biocomposites were fixed on a metal support with carbon tape and metalized with gold in a Balzers Union SCD 030 system. This ensured the accurate scanning of secondary electrons during microscopy using a microscope scanner JSM-IT200 (Tokyo, Japan), operating at 10 kV to 50 µm zoom. SEM images were acquired using the proprietary JEOL software (Akishima, Japan). The functional groups of the biocomposites were investigated using Fourier transform infrared spectroscopy (FTIR) performed in a Bruker equipment (Tensor 27 model), using an attenuated total reflectance (ATR) accessory. The spectra were obtained in the 4000 to 600 cm^−1^ spectral region, with a resolution of 4 cm^−1^, and acquired with 32 scans. Samples were also analyzed on a Rigaku MiniFlex 600 Diffractometer (Tokyo, Japan) equipped with a copper tube operated at 40 mA and 45 kV. Scanning was performed between 5° and 100° in steps of 0.03°/2θ, every 3 °C.min^−1^, to analyze the phase transformations, chemical composition, and crystalline structure of the specimens.

### 2.6. Data Analysis 

Data were subjected to normality (Shapiro–Wilk) and homoscedasticity (Bartlett) tests. Analysis of variance was carried out following a completely randomized design, with seven replicates, with three response variables related to the final temperature of charcoal pyrolysis (400, 600, and 800 °C). After detecting significant differences, the regression model that best predicted the behavior of the data was adjusted. All analyses were performed at 95% probability. Dispersion measures (standard error) were provided to better understand the confidence interval obtained for each studied property. R core Team software version 4.3.0 was used for all statistical analyses. The R core Team software 4.3.0 was used for all statistical analyses.

## 3. Results and Discussion 

### 3.1. Characteristics of Charcoal

**Figure A2:**
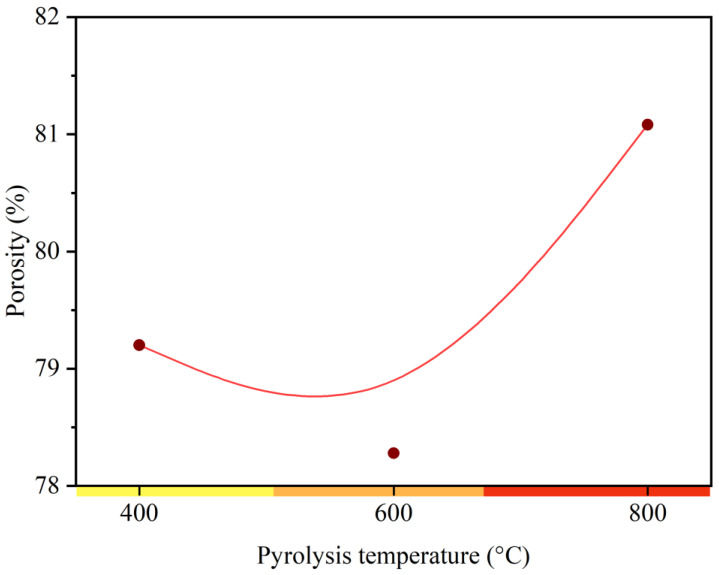
Porosity of charcoal at the pyrolysis temperatures under study.

**Figure A3:**
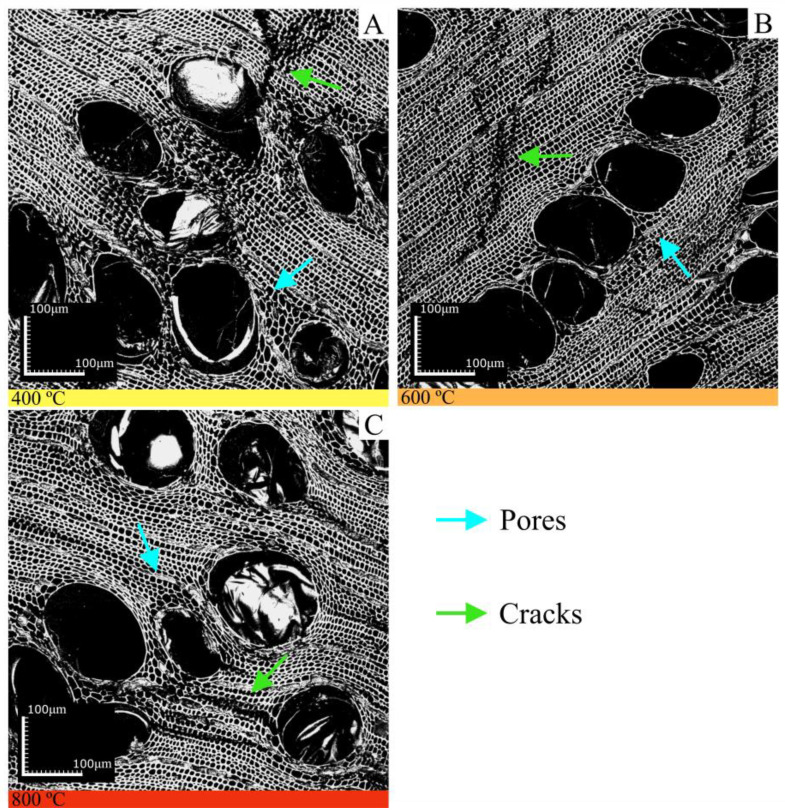
Confocal microscopy of the anatomical structure of charcoal at the pyrolysis temperatures under study. Where: (**A**) Charcoal at 400 °C; (**B**) Charcoal at 600 °C; (**C**) Charcoal at 800 °C.

Porosity is one of the main characteristics present in charcoal that favors its use as filler for the production of biocomposites. Considered a highly porous material, the volume of charcoal can contain up to 85% pores, varying sizes, depending on the raw material and pyrolysis temperature [28,29]. Figure A2 (Appendix A) elucidates that raising the pyrolysis temperature results in more porous charcoal, possibly due to a more intense removal of volatile materials present in the charcoal pores [30]. The pyrolysis temperature of 800 °C (96.77%) further favored the formation of a porous structure, promoting better adhesion with the polymer matrices and, consequently, greater mechanical resistance of the biocomposites to be produced. The Confocal images (Figure A3—Appendix A) at 800 °C were marked by large numbers of pores. This characteristic is relevant for using the material as a bioreinforcement, favoring better adhesion with polymer matrices.

**Figure A4:**
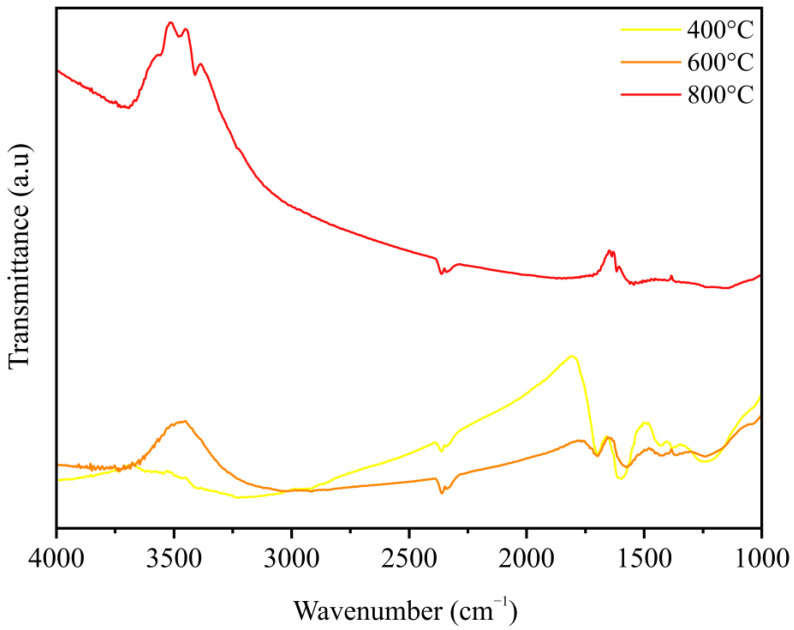
FTIR spectra of charcoal at the pyrolysis temperatures under study.

The FTIR spectra of the materials (Figure A4—Appendix A) elucidated that the peaks related to the stretching vibration of the asymmetric OH group, referring to the phenol, alcohol, and carboxylic acid groups and water (3663 cm^−1^), those referring to vibration and elongation of the CO double bond (1671 and 1728 cm^−1^), and those referring to double bonds between aromatic carbons with olefin and aromatic structures (1500 cm^−1^), were affected by increasing the pyrolysis temperature. The temperature of 800 °C favored the formation of hydroxyl groups, which may have favored the interaction with the polymeric matrix since its polarity was high [31]. 

**Figure A5:**
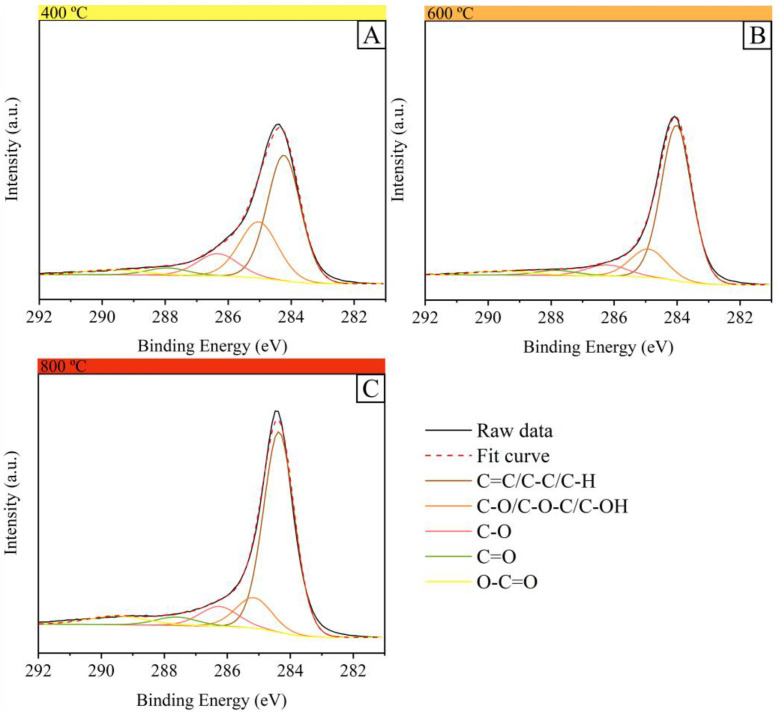
XPS spectra of charcoal at the pyrolysis temperatures under study. Where: (**A**) Charcoal at 400 °C; (**B**) Charcoal at 600 °C; (**C**) Charcoal at 800 °C.

Polarity is an important chemical property present in the material used for the production of biocomposites, as it favors mechanical properties [32]. The changes in the structure and chemical nature caused by the increase in the final pyrolysis temperature of the charcoals are also confirmed by the increase in the intensity of the peaks attributed to the aromatic/aliphatic groups in the XPS spectra (Figure A5—Appendix A). With the increase in the final pyrolysis temperature, it is clear that the energy of the C-C/C-H group dominates the composition of charcoal, which can be classified as hydrophobic [33,34,35,36]. Materials with greater hydrophobicity ensure better compatibility with polymers, which favors the use of charcoal with higher pyrolysis temperatures to be used for the production of biocomposites [32].

### 3.2. Biocomposites from Charcoal

**Figure 2:**
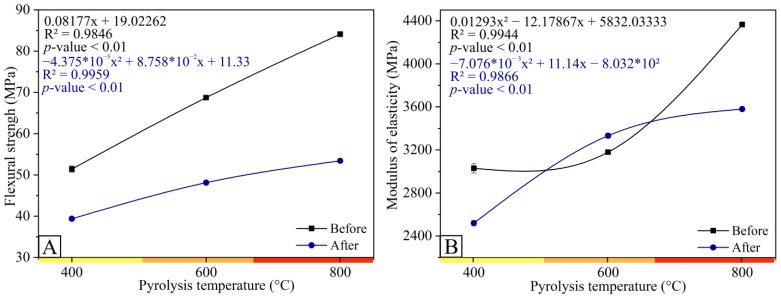
Flexural properties of biocomposites produced with charcoal at different temperatures before and after photoluminosity: (**A**) flexural strength and (**B**) modulus of elasticity.

The effect of pyrolysis temperature and photoluminosity on biocomposites reinforced with charcoal flexural strength properties are shown in Figure 2A,B. The composite without charcoal (control treatment) showed a resistance of 83.24 MPa. After the application of photoluminosity, there was a decrease of 53% in flexural strength. It is different from the modulus of elasticity, which presents a resistance of 2399.70 MPa, with an increase in resistance of 8% after photoluminosity, thus favoring the use of the biocomposite produced with charcoal for structural purposes.

It is noticeable that the flexural properties of the biocomposites, both in terms of tensile strength and modulus of elasticity, decreased after photoluminosity, with a decrease of 5%, 23%, 43%, and 57% for the flexural strength in the composite without fines (control treatment) and in biocomposites, at temperatures of 400, 600, and 800 °C, respectively.

This behavior can be attributed to the decrease in adhesion of the fines/matrix, the weakening of interfacial bonds, and structural changes in the macroscale of the biocomposite. The same behavior occurred with bagasse fibers demonstrated by Lila et al. (2019) [37]. Studies on photoluminosity explain that the loss of mechanical performance after this can be linked to a combination of several mechanisms, such as the predominant degradation of the amorphous structure, plastification caused by moisture absorption, and swelling stresses induced by the difference in expansion and contraction in the fines and polymer matrix [38]. On the other hand, when changing the fines used in the production of biocomposites, those obtained at 400 to those obtained at 800 °C, the modulus of elasticity (Figure 2A) shows a trend in its behavior, decreasing in the initial stages of exposure (3029, 37 MPa) and increasing at a temperature of 600 °C (3179.63 MPa) and 800 °C (4364.3 MPa), showing superior resistance in the biocomposite produced at a higher temperature (800 °C).

**Figure 3:**
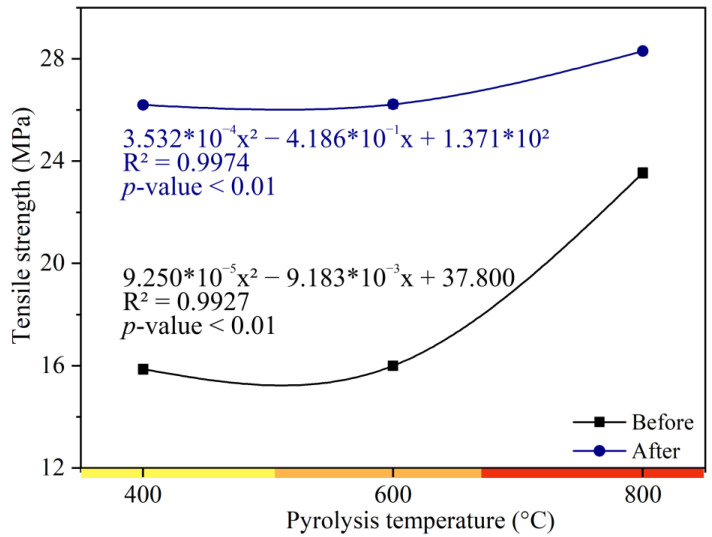
Tensile properties of charcoal biocomposites before and after photoluminosity.

The control treatment showed tensile strength of 51.59 MPa and 20.19 MPa, before and after photoluminosity, respectively. The tensile strength of the biocomposites with charcoal (Figure 3) after photoluminosity obtained an increase of 69.24%, 68.98%, and 54.38% in the biocomposites at 400, 600, and 800 °C, respectively, in relation to the control treatment. The photoluminosity process increased the tensile strength of the biocomposites at temperatures of 400 (22%), 600 (23%), and 800 °C (28%) compared to the control treatment.

This behavior supports that, since photoluminosity tends to reduce the tensile strength of biocomposites, the addition of charcoal obtained with higher pyrolysis temperatures (800 °C) is beneficial for obtaining a more structurally resistant material (Figure 2 and Figure 3), minimizing this loss of resistance, corroborating with trends of carbonaceous materials found in the literature [8]. Carbon filler is a relevant component of polymeric biocomposites due to its favorable properties and modification possibilities which, in combination with suitable polymeric matrices, has a positive effect on the mechanical properties and resistance of biocomposites to environmental agents and UV-C radiation [14]. The mechanical performance of biocomposites after photoluminosity depends on the volumetric fraction of the fines, the level of dispersion, and mainly the state of interfacial adhesion [38,39,40].

**Figure 4:**
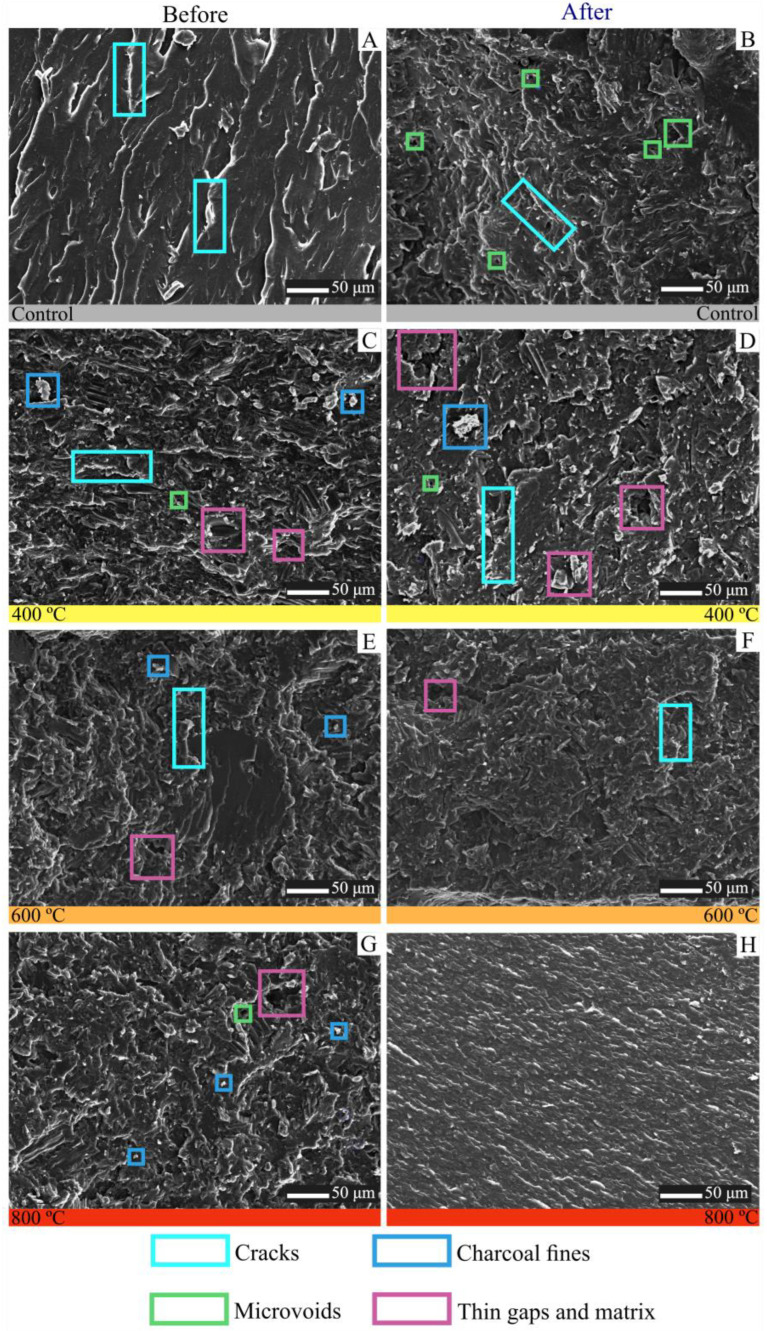
Microstructure of charcoal-reinforced polyester matrix biocomposites. (**A**) composite before photoluminosity; (**B**) composite after photoluminosity; (**C**) biocomposites with charcoal obtained at 400 °C before photoluminosity; (**D**) biocomposites with charcoal obtained at 400 °C after photoluminosity; (**E**) biocomposites with charcoal obtained at 600 °C before photoluminosity; (**F**) biocomposites with charcoal obtained at 600 °C after photoluminosity; (**G**) biocomposites with charcoal obtained at 800 °C before photoluminosity; (**H**) biocomposites with charcoal obtained at 800 °C after photoluminosity. µm = micrometer.

The microstructures of biocomposite surfaces are shown in Figure 3. Composites without fines (control treatment) showed a larger number of cracks and a rougher surface after the photoluminosity process (Figure 4A,B). This roughness can also be observed for biocomposites reinforced with carbon fines obtained at a pyrolysis temperature of 400 °C, making evident the low interaction between the fines and the polyester resin (Figure 4C,D), negatively influencing the resistance to flexion (Figure 2) and traction (Figure 3). However, when analyzing the biocomposites made with charcoal obtained at a higher final pyrolysis temperature (600 and 800 °C), it is noted that the roughness tends to decrease and the interaction between the fines and the polyester resin increases when subjected to the process of photoluminosity (Figure 4E). These observations corroborate the results obtained in the mechanical tests (Figure 2 and Figure 3).

When comparing the flexural strength before and after the photoluminosity, it is verified that the resistance decreased with the effect of the photoluminosity process. This decrease may be due to exposure to UV-c radiation, which can affect the polymer structure of the material, preventing the polymer molecular chains from diffusing and migrating to the surface of the polymer growth face [41,42,43]. Large amounts of cracks are notorious (Figure 4), which may favor the rupture of the biocomposites in a central way, a behavior shown in flexural strength (Figure 2).

It is observed that as the pyrolysis temperature increases, the biocomposites exposed to photoluminosity have fewer microvoids, cavities, and gaps between the fines and the matrix. As a result of the photoluminosity process, it is observed that cavities and microvoids in the fracture surfaces of biocomposites decrease the interfacial bond between bioreinforcement and polymer. As indicated by Mendes et al. [43], the hydrophobic nature of bioreinforcements makes them compatible with hydrophobic thermoplastic polymers, such as polyester resin, revalidating the behavior found in XPS (Figure A5—Appendix A). The spaces formed are one of the parameters that express the quality of the interfacial connection between the fines, and the polymeric matrix. As the number of gaps increases, the quality of the interfacial bond decreases [41,42,43,44,45,46,47,48,49,50]. In this context, when biocomposites containing fines obtained at higher pyrolysis temperatures are analyzed, the smaller the number of gaps observed between the fines and the matrix subject to photoluminosity.

**Figure 5:**
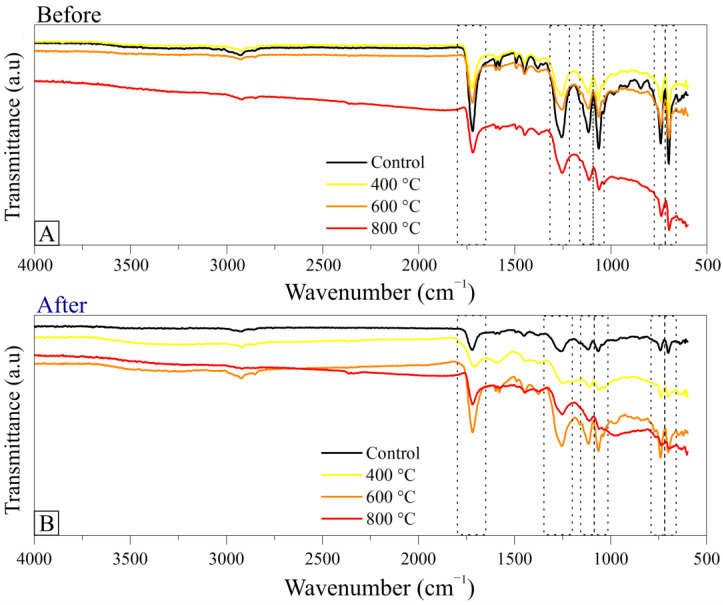
FTIR spectra of the composite without charcoal (control treatment) and biocomposites with charcoal obtained at different pyrolysis temperatures (**A**) before photoluminosity and (**B**) after photoluminosity.

The biocomposite’s chemical changes in the control treatments provided by the photoluminosity, were investigated by the FTIR technique. Figure 5 shows the FTIR spectra of the resin without adding charcoal and the composites without fines (control treatment).

**Figure A6:**
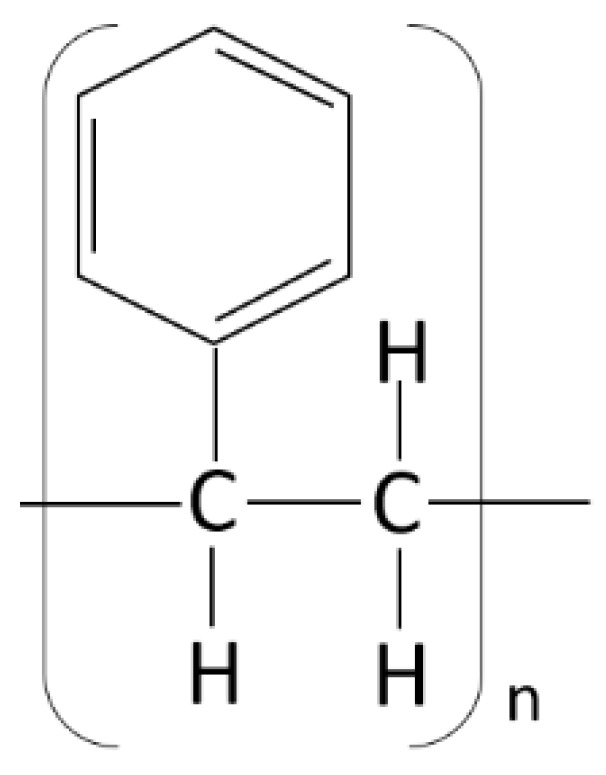
Chemical structure of polyester (C_8_H_8_)_n_.

The chemical structure of polyester resin contains an aromatic ring attached to a carbon of the aliphatic hydrocarbon chain of the monomer unit (Figure A6—Appendix A). As expected, the FTIR spectrum referring to the material without the addition of carbon fines has a typical profile of the polystyrene polymer, with bands in the region between 3060–2800 cm^−1^, which are characteristic of the absorption of aliphatic and aromatic C-H stretching vibrations mode. The absorption band located at 1720 cm^−1^ corresponds to the C=O stretching vibration, which may be present in regions of the polymeric chain due to the use of organic peroxide as a radical initiator (crosslinking initiator) during the polymerization reaction. Still, in the FTIR spectrum of the resin, the absorption bands present between 1600 and 1450 cm^−1^ are attributed to the C=C stretching vibration in aromatic compounds. The aliphatic C-H bending is observed in the absorption band at 1375 cm^−1^. C-O stretching vibration absorption bands appear at 1250 cm^−1,^ and in the region between 1150 and 1050 cm^−1^. The absorption bands attributed to the out-of-plane C-H bending vibrations present in the ring appear between 900 and 675 cm^−1^ [51].

Photoluminosity can lead to changes in the chemical structure of the resin, such as the abstraction of hydrogen atoms from the polymeric chain, forming unsaturated groups [51], or the formation of free radicals [52], among others. These changes in the structure of the molecule can result in changes in the FTIR profile, mainly in the absorption region due to the presence of carbonyl groups (1830–1600 cm^−1^) and C-H (~3500 cm^−1^) [43]. Usually, the absorption band located around 1720 cm^−1^ may convolve into more absorption bands as photoluminosity progresses. Furthermore, the appearance of absorption bands in the region of 3500 cm^−1^, or their increase in intensity, would confirm the effect of photoluminosity on the polymeric chain [53]. However, these aspects were not seen in the FTIR spectra of the photodegraded polymeric resin investigated in this work. On the other hand, the absorption bands located at 988 and 652 cm^−1^ (out-of-plane C-H bending vibrations) shifted to lower wavenumbers, probably due to the influence of structural changes in the photodegraded polymeric chain, such as conjugation effects [51].

The FTIR spectra of the composites showed similar profiles to the polyester spectrum, indicating that there were no significant structural changes in the composites that could be detected by photoluminosity. However, some absorption bands changed their intensities after the incorporation of charcoal in the resin, probably caused by the filling of the spaces distributed along the polymeric matrix by charcoal particles. Furthermore, the charcoal added to the resin can interact with the polymer via π–π interactions between the graphitic structure and the aromatic units of the polyester, also causing this variation in the magnitude of some spectral bands [20,23,38,39].

**Figure 6:**
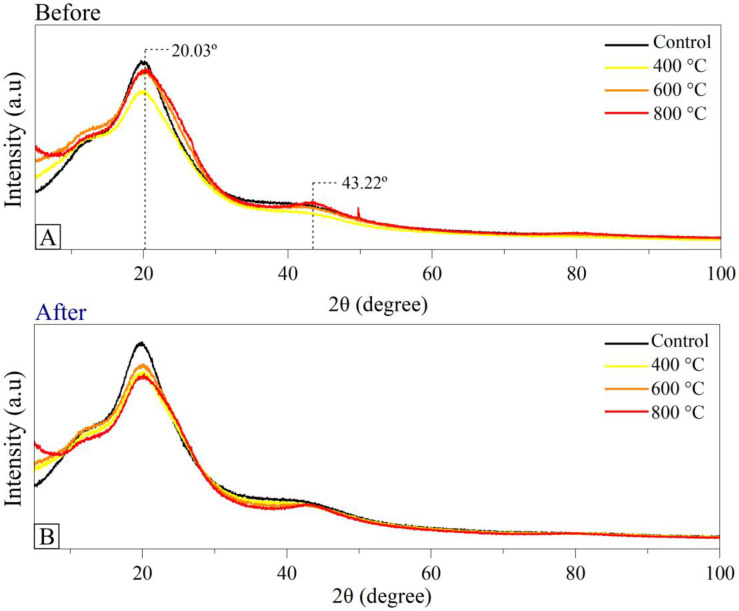
X-ray diffractograms of composites without charcoal (control treatment) and biocomposites with charcoal obtained at different pyrolysis temperatures developed (**A**) before photoluminosity and (**B**) after photoluminosity.

This phenomenon was more evident in the FTIR spectra of the composites containing charcoal obtained at the highest pyrolysis temperatures (600 and 800 °C), which have better organization of their chemical structure promoted by the increase in the pyrolysis temperature. The more organized structure of charcoal results in materials with better hydrophobicity and, therefore, with a greater tendency to disperse uniformly in the intercrossed polymeric chain, thus reducing the gaps formed in the polymerization process [54]. These characteristics can also be evidenced by the variation in the intensity of the absorption peaks observed in the spectra of the composites [55]. Figure 6 shows the X-ray diffractograms of the composites before and after photoluminosity. 

A typical diffraction profile of amorphous materials with high carbon content in the composition is observed (Figure 6), which can be seen by the presence of two broad peaks centered on 2θ de ~20° e 43° [56]. X-ray diffractograms did not show significant changes in the structure of biocomposites after ultraviolet irradiation. However, a slight increase in peak intensity close to 20° was observed in all composites, probably due to the greater structural ordering of the polymeric chain caused by the photoluminosity process.

## 4. Practical Applications and Future Perspective 

The results obtained in this work show promising results on the evaluation of resistance to exposure of biocomposites to UV-c radiation. With this information, it is possible to establish durability parameters of the material according to its exposure to weather conditions. The charcoal fines showed significant results, indicating that the presence of these bioreinforcements, especially those produced at higher temperatures (800 °C), reduce the negative impacts of UV-c exposure on the mechanical, chemical, and thermal properties of the biocomposites. Such characteristics can be considered as promoting increased durability of biocomposites, which can enable their use for different applications that demand a series of structural efforts and varied exposure to climatic and environmental conditions. With more information about the resistance and durability of biocomposites exposed to photoluminosity conditions, it will be possible to direct, in a more straightforward way, these materials to the civil construction and automotive industries, which demand high resistance of materials to environmental exposures.

## 5. Conclusions

The pyrolysis temperature and the demonstrated porosity is of significant relevance, as higher temperatures result in a more porous structure, favoring more effective interactions with the polymer matrices. An in-depth porosity analysis revealed that higher pyrolysis temperatures, notably those around 800 °C, led to the formation of a more porous carbon structure, thus increasing the mechanical strength of the biocomposites. In this context, the mentioned temperature plays a crucial role in promoting the generation of hydroxyl groups, which optimize adhesion to polymer matrices. The observations related to the effect of photoluminosity on the mechanical strength of the material further deepen our understanding. Although photoluminity initially had a negative impact on mechanical strength, the incorporation of charcoal from higher pyrolysis temperatures showed a substantial increase in mechanical strength after exposure. As a result of the photoluminosity process, it is observed that cavities and microvoids in the fracture surfaces of biocomposites decrease the interfacial bond between bioreinforcement and polymer. The hydrophobic nature of the bioreinforcements makes them compatible with hydrophobic thermoplastic polymers such as polyester resin, revalidating the behavior found in XPS. This phenomenon was more evident in the FTIR spectra of the composites containing charcoal obtained at the highest pyrolysis temperatures (800 °C), which show better organization of their chemical structure promoted by the increase in the pyrolysis temperature. The more organized structure of charcoal results in materials with better hydrophobicity and, therefore, with a greater tendency to disperse evenly in the polymer chain, thus reducing the gaps formed in the polymerization process.

Finally, these findings have a significant impact on the durability of carbon-reinforced biocomposites, particularly those from higher pyrolysis temperatures. This behavior provides a promising path for applications in demanding sectors such as civil and automotive engineering, where materials that resist these environmental conditions are required.

## 6. Patents

In this article, an innovative methodological approach aimed at the effects of photoluminosity in biocomposites is presented. The objective is to obtain a deeper understanding of their behavior in order to improve the properties and expand the application possibilities of these materials. The legal protection of this innovation is ensured through the patent process number BR 10 2022 026841 0.

## Data Availability

Not applicable.
